# Nuclear receptor complement of the cnidarian *Nematostella vectensis*: phylogenetic relationships and developmental expression patterns

**DOI:** 10.1186/1471-2148-9-230

**Published:** 2009-09-10

**Authors:** Adam M Reitzel, Ann M Tarrant

**Affiliations:** 1 Department of Biology, Woods Hole Oceanographic Institution, Woods Hole, MA 02543 USA

## Abstract

**Background:**

Nuclear receptors are a superfamily of metazoan transcription factors that regulate diverse developmental and physiological processes. Sequenced genomes from an increasing number of bilaterians have provided a more complete picture of duplication and loss of nuclear receptors in protostomes and deuterostomes but have left open the question of which nuclear receptors were present in the cnidarian-bilaterian ancestor. In addition, nuclear receptor expression and function are largely uncharacterized within cnidarians, preventing determination of conserved and novel nuclear receptor functions in the context of animal evolution.

**Results:**

Here we report the first complete set of nuclear receptors from a cnidarian, the starlet sea anemone *Nematostella vectensis*. Genomic searches using conserved DNA- and ligand-binding domains revealed seventeen nuclear receptors in *N. vectensis*. Phylogenetic analyses support *N. vectensis *orthologs of bilaterian nuclear receptors in four nuclear receptor subfamilies within nuclear receptor family 2 (COUP-TF, TLL, HNF4, TR2/4) and one putative ortholog of GCNF (nuclear receptor family 6). Other *N. vectensis *genes grouped well with nuclear receptor family 2 but represented lineage-specific duplications somewhere within the cnidarian lineage and were not clear orthologs of bilaterian genes. Three nuclear receptors were not well-supported within any particular nuclear receptor family. The seventeen nuclear receptors exhibited distinct developmental expression patterns, with expression of several nuclear receptors limited to a subset of developmental stages.

**Conclusion:**

*N. vectensis *contains a diverse complement of nuclear receptors including orthologs of several bilaterian nuclear receptors. Novel nuclear receptors in *N. vectensis *may be ancient genes lost from triploblastic lineages or may represent cnidarian-specific radiations. Nuclear receptors exhibited distinct developmental expression patterns, which are consistent with diverse regulatory roles for these genes. Understanding the evolutionary relationships and developmental expression of the *N. vectensis *nuclear receptor complement provides insight into the evolution of the nuclear receptor superfamily and a foundation for mechanistic characterization of cnidarian nuclear receptor function.

## Background

**Figure 1:**
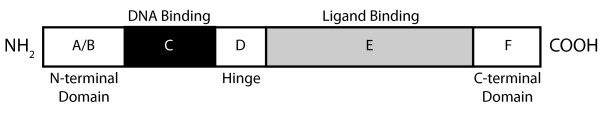
**Nuclear receptor gene structure**. Nuclear receptors are composed of two well-conserved regions, the DNA binding domain (DBD) and the ligand binding domain (LBD).

Nuclear receptors (NRs) are one of the most abundant classes of transcription factors in metazoans and coordinate diverse processes ranging from developmental patterning to physiological regulation. NRs appear to be restricted to animals because extensive work has shown nuclear receptors are present in all metazoan phyla (e.g., sponges [1,2], cnidarians [3,4], and bilaterians [5]) but not in plants, fungi or choanoflagellates [6]. The signature motifs of NRs are the DNA-binding domain (DBD), which includes a Cys-Cys zinc coordinating region, and the ligand-binding domain (LBD), a carboxy-terminal domain that binds ligands and facilitates receptor dimerization and coactivator recruitment (Figure 1). High sequence conservation within these domains, particularly the DBD, has permitted identification of NRs from a number of animals and phylogenetic analysis of the NR superfamily [5].

Genes in the NR superfamily have been classified into six major NR families, many of which are further divided into subfamilies of orthologs and paralogs. All six families are represented in both the protostome and deuterostome lineages, supporting the hypothesis that NRs had undergone extensive radiation prior to the divergence of triploblastic animals. An analysis by Bertrand et al. [5] of NRs from nine bilaterian genomes did much to resolve the evolutionary history of this superfamily. They inferred 25 NRs that likely existed in the Urbilaterian with at least one gene from each of the six families. After this divergence, many NRs underwent extensive duplication in the vertebrates, particularly fishes, and one gene underwent extensive duplication in nematodes [7]. With the increasing availability of sequenced genomes and PCR-based surveys of NR diversity, particularly from non-ecdysozoan protostomes [8-10], it has become apparent that a number of NRs have also been lost since the Urbilaterian ancestor, particularly in insects and nematodes.

Because the NR superfamily had already diversified prior the divergence of the protostomes and deuterostomes, it is essential to characterize NRs in "basal" metazoans to understand not only when the NR families evolved but also the evolutionary relationships among these families. NRs have been reported from a handful of cnidarians from three of the four classes (Anthozoa [4], Cubozoa [11], Hydrozoa [3]) and two sponges [1,2]. Sequence and phylogenetic analyses of identified NRs have led these authors to suggest that many or all of these genes belong to NR family 2 with homologs of TLL (tailless, NR2E) in corals [4], HNF4 (hepatocyte nuclear factor 4, NR2A) in coral [4] and sponge [1], RXR (retinoic × receptor, NR2B) in the box jelly [11], and COUP-TF (chicken ovalbumin upstream promoter transcription factor, NR2F) in coral and hydra ([3,4]).

Despite the importance of understanding how NR regulation of development and physiology has evolved, little has been reported regarding the expression and function of NRs in non-bilaterian animals. Some cnidarian NRs have been shown to share conserved features with their vertebrate orthologs. For example, the RXR homolog in the box jelly *Tripedalia cystophora *specifically binds 9-*cis *retinoic acid with an affinity similar to that of vertebrate RXRs, supporting a conserved function of RXR in these evolutionary distant taxa [11]. The COUP-TF homolog from hydra is expressed in neuronal cells, suggesting an evolutionary origin in nerve cell specification [3]. Many of the identified NRs in cnidarians are orphan receptors (lacking known ligands). Cnidarian tissues contain diverse lipids including prostaglandins, fatty acids, sterols and steroids, and it is plausible that some of these compounds act as ligands for nuclear receptors [12-18]. In addition, the developmental expression of most cnidarian NRs remains uncharacterized. Temporal patterns of expression can provide significant insight into the potential role for NRs during development and metamorphosis as well as the opportunity for co-expressed NRs to interact with one another [19].

Here we present the first genomic analysis of nuclear receptors in the starlet sea anemone, *Nematostella vectensis*, and quantify expression of all identified NRs throughout the life cycle. We identified seventeen nuclear receptors in the genome of *N. vectensis*. Many of the NRs did not show clear orthology to bilaterian NRs, and some *N. vectensis *NRs appear to have resulted from lineage specific gene duplications. Expression of most NRs varied significantly during development, with clusters of NRs exhibiting similar expression patterns.

## Results

### Phylogenetic relationships of *N. vectensis *nuclear receptors

We identified 17 NRs from *N. vectensis *with a combination of bioinformatic searches of the assembled *N. vectensis *genome [20]. We have designated these as NvNR1-17 (Accession Numbers Table 1, numbering is arbitrary and does not indicate phylogenetic position within the NR superfamily; for amino acid sequences see Additional file 1). All of these genes were amplified from cDNA preparations and confirmed as expressed transcripts.

**Table 1:** Accession numbers of *N. vectensis *nuclear receptors from NCBI GenBank, JGI *Nematostella *Database, and ESTs (where available).

**Gene Name**	**GenBank Acc**.	**JGI Gene Model**	**Matching ESTs**
NvNR1	XP_001634258	101676	NA
NvNR2	XP_001636937	99425	NA
NvNR3	XP_001632045	108851	JGI_CAAB2572.fwd
NvNR4	XP_001638550	89471	JGI_CAAD1662
NvNR5	XP_001630386	114090	JGI_CAGN9558.fwd
NvNR6	XP_001635112	183874	JGI_CAGN4016
NvNR7	XP_001630385	169225	JGI_CAGN3217.fwd
NvNR8	XP_001634999	99425	NA
NvNR9	XP_001624815	247458	JGI_CAGG3386.fwd
NvNR10	XP_001629708	189134	JGI_CAAB5823
NvNR11	XP_001634340	242271	JGI_CAGN16932
NvNR12	XP_001634378	165424	JGI_CAGH2972
NvNR13	XP_001636010	203423	NA
NvNR14	XP_001636637	202735	NA
NvNR15	XP_001631902	167880	JGI_CAGH3462.fwd
NvNR16	XP_001631058	244121	JGI_CAGN9351
NvNR17	XP_001624292	218255	JGI_CAGG4622.fwd

**Figure 2:**
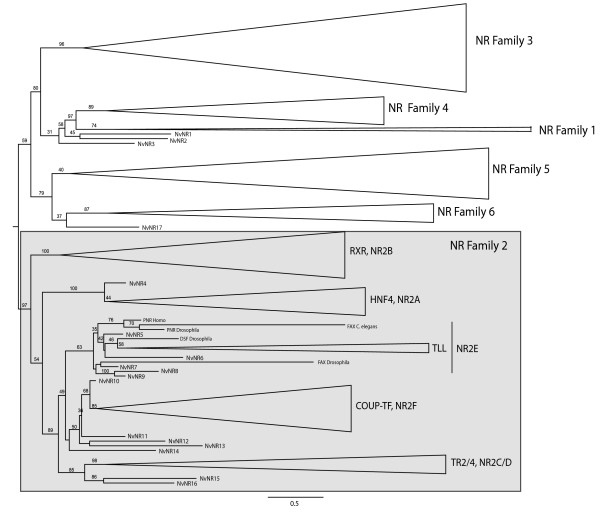
**Phylogenetic relationships of *N. vectensis *NRs (NvNRs) depicted by a maximum-likelihood tree with percent bootstrap support of 1000 bootstraps for nodes of evolutionary significance**. The alignment was constructed using the DBD and a portion of the LBD (additional details in text). Monophyletic relationships for bilaterian sequences in particular families or subfamilies were recovered in all cases but one (NR2E) and are depicted as horizontal triangle. A full tree with all taxa depicted as individual branches is presented in Additional File 2. The tree was rooted with NR family 2 because this family is monophyletic, represents a defined nuclear receptor family, and includes what is likely the original nuclear receptor, represented by an HNF4-like homolog from the sponge *Amphimedon queenslandica *[1]. Most *N. vectensis *NRs group within NR family 2 and many (e.g., NvNR11- NvNR14) are supported as independent radiations of subfamilies within this family. *N. vectensis *also has a NR related to GCNF (NR family 6), but with low support. Three NRs did not group with any previously described family (NvNR1-3). These genes may represent ancestral genes that later diversified into one or more of the NR 1, 3, and 4 families.

When DBD plus LBD were used for phylogenetic analyses of *N. vectensis *and bilaterian NRs, the topology of the tree with the highest likelihood was consistent with monophyletic relationships of the recognized NR families. Nodes for NR families 1-4 were each supported by high bootstrap scores (Figure 2, for a more detailed tree with nodes expanded see Additional file 2). Although NR families 5 and 6 were each weakly supported (BS = 40 and 37, respectively), a clade grouping these two families was fairly well supported (BS = 79) as previously shown by Thornton [21]. Phylogenetic analyses using just the DBD or portion of the LBD resulted in more poorly supported nodes throughout the tree and in some cases failed to recover monophyletic NR families (see Additional files 3 and 4). In addition, a complementary analysis using neighbor joining with the DBD and LBD also resulted in weakly supported nodes for the NR families (see Additional file 5). Therefore, we inferred the phylogenetic relationships of the *N. vectensis *NRs from the likelihood analysis based on the DBD plus LBD.

NvNR1, 2, and 3 cannot be unambiguously assigned to a family and nest as equally related to NR families 1 and 4. A majority of *N. vectensis *NRs (NvNR4-16) are strongly supported as homologs of genes in NR family 2. These *N. vectensis *NRs are associated with four subfamilies within family 2. NvNR4 is an ortholog of HNF4 (subfamily 2A), which has also been identified in vertebrates, insects, and nematodes [22,23]. NvNR5-9 cluster with subfamily 2E (TLL, FAX, PNR). NvNR5 and 6 are most closely related to TLL with moderate bootstrap support. NvNR7-9 do not have a clear orthologs with the bilaterian genes from this subfamily and thus may represent a cnidarian-specific radiation. NvNR10-14 group with subfamily 2F (COUP-TFs), and among these, NvNR10 is most closely related to the bilaterian COUP-TFs. NvNR15-16 are supported as homologs of the testicular orphan receptors (TR2/TR4, subfamily 2C/D). An RXR homolog (jRXR, subfamily 2B) has been identified in the box jelly *Tripedalia cystophora *[11], but an RXR homolog is not apparent in the *N. vectensis *genome. NvNR17 groups with NR family 6 (GCNF, germ cell nuclear factor) but with relatively weak support. In the neighbor joining analysis, NvNR17 was positioned as an outgroup to NR families 5 and 6 but with low bootstrap support. A GCNF-like gene has not been previously reported from a cnidarian.

### Gene structure

*N. vectensis *NRs are composed of between 3 and 7 exons and span 2059 to 11741 bp of genomic sequence (see Additional file 6). Because we currently have complete transcript sequence with 5- and 3- prime untranslated region (UTR) for fewer than half of the NRs (6 of 17), additional exons may be identified when complete transcripts are confirmed. In some cases (e.g., NvNR10), exons were composed completely of UTR sequence; thus it is likely that additional exons will be identified for some of the NRs with uncharacterized UTR sequence.

Phylogenetic analyses indicated that several NRs in *N. vectensis *are most closely related to one another suggesting potential lineage-specific duplication. With the exception of two genes (NvNR11 and NvNR12), the NRs in *N. vectensis *are located on different scaffolds. Thus, the NR diversity in *N. vectensis *does not appear to have resulted from recent tandem duplications, but instead may represent more ancient duplications within the cnidarian lineage. Some closely related genes also shared conserved intron-exon structure (see Additional file 7). For example, NvNR8 and NvNR9 group together with 100% bootstrap support and share two of three splice sites.

### Developmental expression of nuclear receptors

**Figure 3:**
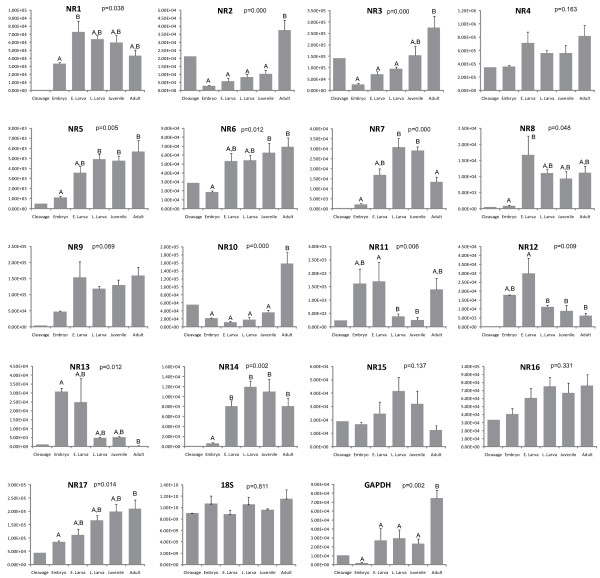
**Developmental expression of *Nematostella vectensis *nuclear receptors (NR1-NR17) and putative housekeeping genes (18S and GAPDH)**. Expression measured using qPCR, as described in text. Expression in molecules per μl cDNA indicated on the y-axis. Bar indicate the mean ± standard error of 3-5 biological replicates. The first bars ("cleavage") represents a single sample, which was not included in statistical analysis. Expression patterns were analyzed with a one-way ANOVA followed by pairwise comparisons with Tukey's Honestly Significant Difference Test. Letters indicate groups that were different at a level of p = 0.05. GAPDH showed variable expression over the sampled stages and was not used as a standard for comparing gene expression of NRs among developmental stages.

**Figure 4:**
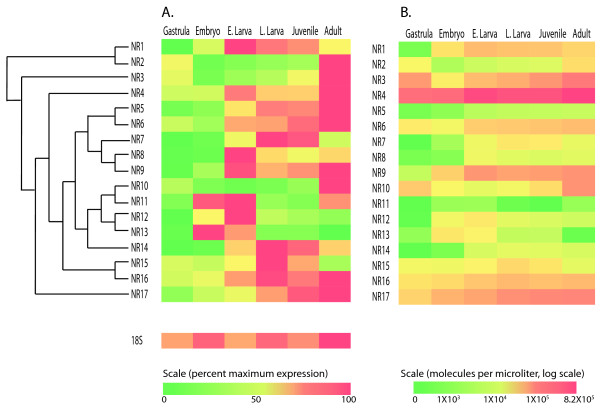
**Heat maps indicating developmental expression of *Nematostella vectensis *nuclear receptors**. Cladogram indicates the topology from Figure 2 with other taxa removed. (A) For each gene, expression is shown on a linear scale as a percentage of the stage with the highest expression. (B) Expression is shown on a log scale as molecules per microliter, calculated in relation to standard curves constructed from serially diluted plasmids.

One-way ANOVA indicated that 13 of the 17 NRs varied significantly in their level of expression during development (Figure 3). Even with relatively coarse developmental staging, we observed clear temporal patterns of gene expression (Figure 4a). NvNR8, 11, 12 and 13 were primarily expressed during embryonic and/or early larval stages with little expression in the juvenile and adult stages. NvNR1, 7, 14 and 15 were most highly expressed during larval and juvenile stages. NvNR4, 5, 6, 9, 16, and 17 showed increased expression during larval and juvenile stages and continued high expression in adults. Finally, NvNR2, 3, and 10 were primarily expressed in adults, and NvNR10 was only minimally expressed in earlier stages. 18S expression did not vary significantly across the stages and is apparently a good housekeeping gene with respect to *N. vectensis *development. We also tested GAPDH as a potential control gene to standardize gene expression. GAPDH expression was highly variable across these developmental stages and was not used for comparisons of NR expression among stages.

Absolute levels of gene expression, normalized to plasmid standards, indicated large differences in number of expressed transcripts among genes (Figure 4b). Although its expression was developmentally variable (Figure 3, 4a), NvNR4 was the most highly expressed NR over all stages (Figure 4b). NvNR3, 9 and 17 also showed high levels of overall expression.

In several cases, phylogenetically closely related genes showed divergent temporal expression patterns during development. For example, NR11 was expressed at very low levels (Figure 4b), primarily during embryonic and early larval stages (Figure 4a). In contrast, NR10 was expressed at moderate levels, mostly in adults.

**Figure 5:**
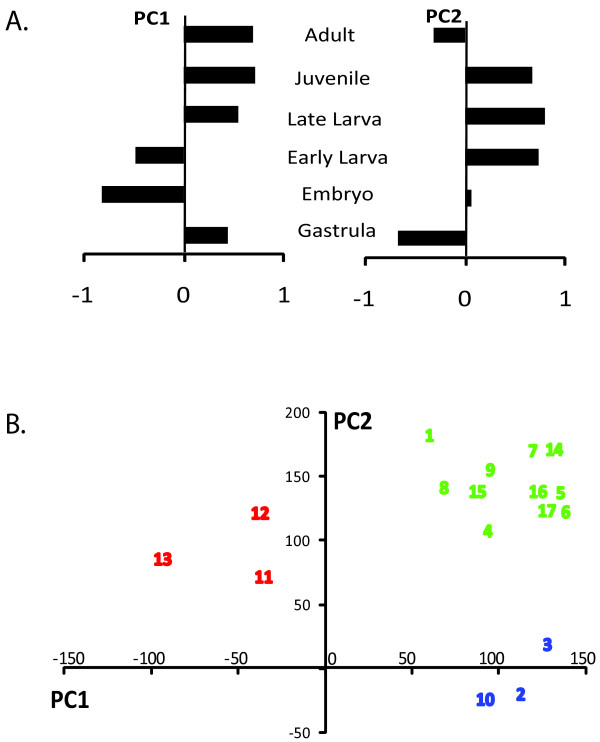
**Principal components analysis of nuclear receptor relative expression data**. Analysis was conducted on gene expression normalized to the maximum expression for each gene, as in Figure 4A. (A) Eigen vectors showing relative contribution of each life stage to each principal component. Longer bars indicate stronger relative contribution. The first and second principal components explained 39% and 36% of the variance, respectively. (B) Scatter plot of first and second principal component scores for all seventeen nuclear receptors. Colors indicate three distinct clusters of NRs showing similar expression patterns. Numbers 1-17 indicate *N. vectensis *nuclear receptors 1-17, respectively.

Principal components analysis (PCA) is a statistical technique for reducing the dimensionality of a dataset, in this case facilitating identification of NRs with similar expression patterns. PCA was conducted for all 17 genes from all developmental stages (Figure 5). The first principal component (PC1) accounted for 39% of the overall variance in gene expression and second principal component (PC2) accounted for 36% of the variance. When the values for PC1 and PC2 are shown on a scatter plot (Figure 5B), three clusters emerged, NvNR11, 12 and 13 clustered to the exclusion of other genes along PC1 and were each highly expressed in early developmental stages (embryo, early larva). A second cluster contained NvNR2, 3, and 10, which are most strongly expressed in adults. The remaining genes constituted a third cluster and were not strongly distinguished from one another by this analysis.

## Discussion

Cnidarians both occupy a key evolutionary position and are ecologically important as predators, prey and structure-builders in marine and freshwater environments. As "basal" metazoans, cnidarians form an outgroup to the bilaterian animals and are intermediate in complexity between sponges and bilaterians. In spite of the acknowledged importance of understanding cnidarians from both an ecological and an evolutionary perspective, cnidarian physiology is poorly understood, particularly at the molecular level. Thus, characterizing cnidarian NRs provides insight both into evolution of NR signaling and bioregulatory processes in a major group of aquatic animals.

### Homology and expression of NvNRs

Analysis of the phylogenetic relationships among NRs from the starlet sea anemone, *Nematostella vectensis*, provides strong support for the diversification of NR family 2 prior to the divergence of the cnidarian and bilaterian lineages. Phylogenetic analyses of *N. vectensis *and bilaterian NRs revealed cnidarian orthologs of HNF4, COUP, TLL, and TR2/4. Grasso et al. [4] identified 10 NRs in the coral *Acropora millepora*, including several members of NR family 2. Our results support their conclusion that family 2 was well diversified prior to the split between the cnidarian and bilaterian lineages.

NvNR4 is an apparent homolog of hepatocyte nuclear factor 4 (HNF4, subfamily 2A), which has also been identified in vertebrates, insects, nematodes, and corals [4,22,23]. In mammals, HNF4α binds endogenous fatty acid ligands [24,25] and regulates hepatocyte differentiation, energetic metabolism and xenobiotic detoxification. HNF4 also regulates insect gut development [26,27]. HNF4 underwent extensive duplications in *C. elegans *[28] such that inferring homologous functions for HNF4 from the nematode is difficult. One *C. elegans *paralog, nhr-49, is involved in energetic metabolism as a regulator of fat storage [29]. In addition, an HNF4 homolog has been cloned from sponges and may be similar to the ancestral NR [1]. Sponge HNF4 is expressed throughout development in ciliated column cells of the outer epithelium [1]. In *N. vectensis*, NvNR4 was expressed at all developmental time points with higher mean expression from larval to adult stages. We predict that NvNR4 will be expressed in endodermal tissue, which would be consistent with conserved roles in development of the digestive epithelium, energetic metabolism, and/or detoxification.

NvNR5- NvNR9 cluster with subfamily 2E (TLL/TLX, FAX, PNR); members of this subfamily have previously been reported in corals [4,30]. In our phylogenetic analyses, we were unable to fully resolve the evolutionary relationships of *N. vectensis *and bilaterian NRs within subfamily 2E (Figure 2). In other animals, members of subfamily 2E are involved in neural differentiation. Tailless homologs in mammals (TLX) and insects (TLL) are particularly important for eye and forebrain development [31-34] and embryonic anterior-posterior patterning [35]. FAX-1 regulates neural patterning in *C. elegans *[36,37], and PNR plays a more specialized role by regulating retinal development in vertebrates [38]. Several *N. vectensis *members of subfamily 2E (i.e., NvNR5, 7, 8) were highly expressed during early developmental stages coinciding with neurogenesis and embryonic patterning [39]. The stage of maximal expression varied among members of this subfamily, indicating potential diversification of function within the cnidarian lineage.

NvNR10-NvNR14 group with subfamily 2F (COUP-TFs); COUP-TFs have also been identified in corals, hydra, flatworms, sea urchin, and lancelets [3,4,40-42]. NvNR10 was most closely related to bilaterian COUP-TFs, while NvNR11- NvNR14 are supported as an independent radiation with this subfamily. COUP-TFs generally act as transcriptional repressors and regulate development of muscles, the heart and the nervous system, particularly differentiation of the hindbrain and photoreceptors [43-46]. In hydra, COUP-TF is expressed in nematoblasts (nematocyte precursors) and in a subset of neuronal cells, consistent with a conserved role of COUP-TF in regulating neural differentiation [3]. COUP-TFs can also affect reproductive processes through cross-talk with estrogen receptors and ecdysteroid receptors in vertebrates and insects, respectively [47-50]. COUP-TF homologs from *N. vectensis *showed diverse expression patterns during development. For example, NvNR12 and13 were primarily expressed during embryonic and/or early larval stages with little expression in the juvenile and adult stages. The expression of these COUP-TF-like genes coincides with neurogenesis and embryo patterning [39]. In contrast, we observed relatively high expression of NvNR10 in adults but lower expression during embryogenesis and larval development.

We identified two members of the subfamily 2C/D (TR2/4, NvNR15-16). TR2/4 homologs have been identified in a range of animals including vertebrates, sea urchins, ascidians, nematodes, insects, flatworms, and corals [51-54]. Protostomes and most deuterostomes have a single TR2/4 homolog. Interestingly, both *N. vectensis *and the coral *Acropora millepora *[4] contain two TR2/4 homologs, suggesting a cnidarian-specific duplication. In general, TR2/4 homologs act as transcriptional repressors through several mechanisms including competition with other nuclear receptors for binding sites and co-factors [55,56]. TR2/4 homologs are broadly expressed in vertebrate tissues [57] and throughout development in mammals [57], ascidians [51], and flatworms [58]. We observed similar ubiquitous expression in *N. vectensis *where both NvNR15 and 16 were expressed throughout all developmental stages.

NvNR17 forms a monophyletic grouping with NR family 6 (GCNFs, germ cell nuclear factors). Although bootstrap support for this assignment is relatively weak, likelihood analyses of the DBD plus LBD and each of these domains individually group NvNR17 as a homolog to the GCNFs. While neighbor joining analysis placed NvNR17 as an outgroup to NR families 5 and 6, bootstrap support for deep nodes of this tree were low relative to the maximum likelihood analyses, potentially obscuring evolutionary relationships. GCNF has been reported from a number of bilaterian phyla. This is the first report of a putative cnidarian homolog from this NR family. Expression of GCNF homologs varies greatly among taxa. In *D. melanogaster*, DmHR4 is expressed following pulses of 20-hydroxyecdysone during discrete stages of embryogenesis, the second larval instar and prepupal development [59,60]. In *C. elegans*, CeNHR-91 is expressed in response to ATP-binding cassette protein E in embryos, larvae and several adult tissues [61]. In vertebrates, GCNF is expressed primarily expressed in gonadal tissues and during embryogenesis [62,63]. In vertebrates, GCNF is involved in neuronal differentiation, germ cell development, and axial patterning, and gametogenesis [63-65]. In our study, NvNR17 expression increased throughout the sampled developmental stages. Highest expression was in adult tissues, which may reflect a role in gametogenesis.

NvNR1-3 did not group well with any previously defined NR family. These genes are most closely related to NR Family 1 and 4. With the available data, we cannot conclude whether these represent the descendents of ancient NRs that later diversified into recognized NR families or if these genes represent an independent radiation somewhere within the cnidarian lineage. Further sampling within the Cnidaria will be necessary to help resolve the evolutionary relationship of these NRs.

We did not identify a member of NR family 3 in *N. vectensis*. NR family 3 is represented in protostomes and deuterostomes [5,21,66,67], and has been more recently identified in the placozoan *Trichoplax adhaerens *[[68], Reitzel and Tarrant, unpublished data] but has not yet been reported from any cnidarian. Thus, NR 3 homologs have apparently been lost early in the cnidarian lineage. NR family 3 contains the vertebrate-type steroid (*i.e*., non-ecdysteroid) receptors. While NR3 family members were not identified in *N. vectensis*, estrogens and other steroids have been detected in cnidarian tissues [12,15,69], are apparently released during spawning events [12,69], and can experimentally affect coral growth and reproduction [70]. The mechanism for steroid action in cnidarians is currently unknown and may be mediated through nuclear receptors that are not orthologs of the vertebrate steroid receptors or through alternative mechanisms.

### RXR loss in *N. vectensis*

*N. vectensis *appears to have lost the ortholog of RXR because this gene is present in another cnidarian, the cubozoan *Tripedalia cystophora *[11]. The RXR homolog (jRXR) from *T. cystophora *bound 9-cis retinoic acid, and heterodimerized with vertebrate thyroid hormone receptor [11]. This evidence suggests that this cnidarian RXR functions in a similar way as vertebrate RXRs. In contrast to the box jelly, an RXR ortholog was not identified in a PCR-based survey of NRs from the coral *Acropora millepora *[4]. However, RXR immunoreactivity has been reported in epithelial nerve tissue of the coral *A. millepora *and the sea pansy *Renilla koellikeri*, but the genes were not identified [71]. Indeed the authors of the immunological study indicated that the epitope for the antibody has highest sequence similarity to *A. millepora *nuclear receptor 8, but phylogenetic analyses indicate that AmNR8 is most similar to TR2/4 [4]. The absence of an RXR ortholog in *N. vectensis *and in corals is particularly interesting from a functional perspective because vertebrate and insect RXRs form heterodimers with many other nuclear receptors to regulate gene expression [48,72-74]. In a parallel example, *C. elegans *independently lost an RXR ortholog, but another NR (nhr-49, HNF4-like) can dimerize with several other NRs [75]. Thus, it has been suggested that nhr-49 may have assumed an RXR-like function as a common heterodimerization partner [76]. Similarly, *N. vectensis *and other cnidarians may have one or more receptors that "promiscuously" form heterodimers with other NRs in a manner similar to bilaterian RXRs. Alternatively, it is possible that RXR has been lost and not replaced with a functional equivalent.

### Gene duplication and divergence

Within NR family 2, our data from *N. vectensis *support independent radiations of several subfamilies. Phylogenetic analyses support two TR2/4 genes and multiple COUP-TF-like NRs in *N. vectensis*. Similarly, we observed 5 *N. vectensis *NRs that group with bilaterian NRs in the subfamily NR 2E. These genes are likely cnidarian-specific radiations within these NR 2 subfamilies and may represent interesting cases for better understanding the diversification of NR function within an early diverging metazoan.

After gene duplications, paralogs may be selected for divergent functions that may be reflected by different expression patterns [77,78]. Previous reports of *N. vectensis *paralogs have suggested that duplicated genes have varying degrees of distinct spatial and temporal gene expression patterns (e.g., snail [79], Otx [80]). In some cases, NvNRs supported as likely lineage specific duplications showed divergent temporal gene expression patterns consistent with subfunctionalization. For example, NvNR1 was highly expressed in the larval and juvenile stages, and the closely related NvNR2 was most highly expressed during cleavage and in adults. *In situ *hybridization studies of *N. vectensis *will reveal spatial expression patterns (e.g., anterior-posterior gradients, neural expression), providing further insight into the potential subfuctionalization of duplicated NRs.

## Conclusion

In this study, we have identified 17 nuclear receptors in the sea anemone *Nematostella vectensis*. Phylogenetic analyses indicate that a majority of these NRs are members of NR family 2 and are consistent with previous studies indicating that this family had diversified prior to the cnidarian-bilaterian split. We also identified a putative homolog to GCNF in NR family 6 as well as three NRs that do not group with any previously identified NR family. Expression of *N. vectensis *NRs varied during development, consistent with stage-specific functions related to development, metamorphosis and adult physiology. Future studies of NR function in *N. vectensis *will provide a critical understanding of the evolution the nuclear receptor superfamily in animals.

## Methods

### Animals

The starlet sea anemone, *Nematostella vectensis*, is a small burrowing anemone found in estuaries along the Atlantic Coast of the United States, as well as in populations in England and along the Pacific Coast of the United States. We collected adults from Great Sippewissett Marsh, MA USA and cultured them under standard conditions (0.45 μm filtered seawater diluted to 13 ppt, room temperature, fed twice weekly brine shrimp and once mussel ovary; similar to previously described conditions [81,82]). Under these conditions, *N. vectensis *undergoes a predictable reproductive cycle that includes weekly spawning. Within 2-3 hours after spawning, embryos were gathered from dishes, rinsed with diluted seawater, and placed in sterile dishes. At specific stages (i.e., cleavage, early planula, late planula [pyramid shape], and juvenile, [81]) we collected individuals with a pipette, lightly centrifuged, decanted excess seawater, and snap froze with liquid nitrogen for later analysis. Adults were left unfed for at least one week prior to freezing to minimize contamination from food sources.

### RNA extraction and cDNA synthesis

For cloning of nuclear receptors, total RNA was extracted from *N. vectensis *of varying developmental stages, ranging from embryos to adult. RNA was extracted by homogenization of whole individuals in STAT-60 (Tel-Test, Inc). RNA for cloning was then pooled from developmental stages. RNA purity and yield were quantified using a ND-1000 spectrophotometer (Nanodrop). RNA quality was visualized for representative samples on a denaturing agarose gel. Polyadenylated-enriched RNA (polyA-RNA) was prepared from total RNA using the MicroPoly(A)Purist Kit (Ambion). Complementary DNA (cDNA) was synthesized from polyA-RNA using the Iscript cDNA Synthesis Kit (Bio-Rad) using 1 μg of polyA-RNA per 20 μl reaction.

For characterization of developmental expression patterns, RNA was extracted using STAT-60 and the Aurum Total RNA Mini Kit with on-column DNAse digestion, as described previously [83]. Each extraction included multiple individuals from a single spawning event or in the case of adults, multiple individuals from the breeding stock. From the total RNA, cDNA was synthesized with the Iscript cDNA Synthesis Kit using 2 μg of RNA per 30 μl reaction. cDNA was prepared from the following developmental stages: cleavage (0.5 days post fertilization [dpf], n = 1), embryos (1 dpf, n = 3), early larvae (3-4 dpf, n = 4), late larvae (7-11 dpf, not metamorphosed, n = 4), juvenile (8-23 dpf, metamorphosed, n = 4), and adult (>100 dpf, n = 4).

### Database searching and cloning

*N. vectensis *nuclear receptors were identified through a combination of BLASTp and domain searches through the Joint Genome Institute *N. vectensis *assembly. Genes were additionally queried against the EST database at JGI in order to annotate more complete transcripts. To confirm the predicted sequences we amplified, cloned, and sequenced large portions of each predicted NR transcript (primers designed with Primer3, primer sequences listed in Table S1). The PCR mixture consisted of 11.8 μl of molecular biology grade distilled water, 0.1 μl of AmpliTaq Gold (Applied Biosystems), 2 μl of the accompanying 10× Buffer, 3.75 mM MgCl_2_, 0.8 mM dNTPs, 0.5 μl of 10 μM gene-specific primers, and 0.5 μl of 1:5 diluted polyA cDNA. PCR conditions were as follows: 95°C for 5 min; 40 cycles of 95°C for 15 s, 60°C for 30 s, and 72°C for 60 s, followed by a 10 min extension at 72°C. Products were gel purified, ligated into pGEM-T Easy vector (Promega), and sequenced to confirm the targeted amplicon.

### Phylogenetic analysis

We used a likelihood based approach to determine evolutionary relationships of the *N. vectensis *NRs with bilaterian NRs. NRs from *Homo sapiens*, *Danio rerio*, *Xenopus laevis*, *Drosophila melanogaster*, and *Caenorhabditis elegans *from Bertrand et al. [5] were used as representative bilaterians. We included sequences for these species from each of the defined families and subfamilies in order to fully represent the broad-scale diversity of NRs. We also included NR sequences from *Ciona intestinalis *reported in [51] and an estrogen receptor homolog from the mollusc *Aplysia californica *[84]. Additional reports of NR diversity from other animals [9,67,85,86] have shown that these taxa fully represent the diversity of NRs to the sub-family level, despite lineage-specific losses in some ecdysozoans and gene-specific duplications in a variety of taxa. Full length sequences for all taxa were aligned with Muscle 3.6 [87] and edited manually in the case of clear errors. Maximum likelihood analyses were run using RAxML (version 7.0.4, [88]) with a JTT+G matrix (model determined by AIC criteria with ProtTest v1.4, [89]). Separate analyses were conducted using the DBD only, a portion of the LDB, and the DBD plus a portion of LBD. We also performed a complementary neighbor joining analysis of the DBD and LBD alignment (PHYLIP v3.6). Trees were visualized and illustrated with FigTree v1.1.2 .

### Gene structure

We assembled the most complete transcripts for each of *N. vectensis*' NRs with a combination of cloned sequence, ESTs, and, where necessary, gene prediction models from the Joint Genome Institute (JGI). Intron-exon structure was determined by aligning the assembled transcripts to the most recent genome scaffolds in the JGI *N. vectensis *database. Gene structure was illustrated with GenePalette v1.21 [90].

### Quantitative real-time RT-PCR (qPCR)

Oligonucleotide primers (see Additional file 8) were designed to amplify each *N. vectensis *NR, as well as glyceraldehyde 3-phosphate dehydrogenase (GAPDH) and 18S ribosomal RNA (18S). Primers were 20-21 nt, with a GC content of 40-60%, either overlapped predicted exon-exon boundaries by 3-4 bp or spanned a large intron, and produced predicted amplicons of 55-146 bp with minimal predicted secondary structure (m-fold, [91]). A standard curve was constructed from serially-diluted plasmids containing the amplicon of interest. The standard curve was used in qPCR reactions to quantify amplification efficiency and to calculate the number of molecules per reaction (as in [92]). qPCR was performed using iQ SYBR Green Supermix (Bio-Rad), and reactions were run a MyCycler Real-Time PCR detection system (Bio-Rad). For each gene, standards were run in triplicate wells and experimental samples were run in duplicate wells (technical replicates) on a single plate. The PCR mixture consisted of 11.5 μl of molecular biology grade distilled water, 12.5 μl of IQ SYBR Green Supermix, 0.5 μl of 10 μM gene-specific primers, and 0.5 μl of cDNA. PCR conditions were as follows: 95°C for 3 min; 40 cycles of 95°C for 15 s and 64°C for 45 s. After 40 cycles, the PCR products from each reaction were subjected to melt curve analysis to ensure that only a single product was amplified. Selected reactions for each gene were visualized on 15% TBE gels (Bio-Rad) and consistently yielded single bands of the predicted size. The number of molecules per μl for each gene was calculated by comparing the threshold cycle (C_t_) from the sample with the standard curve. Expression was compared among developmental stages using one-way analysis of variance (ANOVA) with Tukey's Honestly Significant Difference Test as a posthoc test (SYSTAT 12). Relative gene expression patterns were compared with principal component analysis (PCA, calculated with SYSTAT 12). This statistical approach is similar to that previously described by Tarrant and colleagues [83].

## Authors' contributions

AMR conducted bioinformatic, gene structure, and phylogenetic analysis, designed primers, cloned nuclear receptor fragments, and drafted the manuscript. AMT conceived of the study, conducted qPCR assays and statistical analysis, and contributed to the draft. The authors together interpreted the data and revised the manuscript. Both authors read and approved of the final manuscript.

## Supplementary Material

Additional file 1 ***N. vectensis *deduced amino acid sequences used for phylogenetic analyses**. List of amino acid sequences. Click here for file

Additional file 2 **Maximum likelihood tree using the DBD and portion of the LBD**. NRs were taken from *N. vectensis*, *Homo sapiens*, *Danio rerio*, *Xenopus laevis*, *Drosophila melanogaster*, *Caenorhabditis elegans*, *Aplysia californica*, and *Ciona intestinalis*. Tree was rooted with the NR 2 family. Values above branches indicate percent of 1000 bootstraps. Click here for file

Additional file 3 **Maximum likelihood tree using only the DBD portion of the NR alignment**. Tree was rooted with the NR 2 family. Values above branches indicate percent of 1000 bootstraps. Click here for file

Additional file 4 **Maximum likelihood tree using only the alignable portions of the LBD of the NR alignment**. Tree was rooted with the NR 2 family. Values above branches indicate percent of 1000 bootstraps. Click here for file

Additional file 5 **Neighbor joining tree constructed using the DBD and portion of LDB of representative NRs**. NRs from *N. vectensis*, *Homo sapiens*, *Danio rerio*, *Xenopus laevis*, *Drosophila melanogaster*, *Caenorhabditis elegans*, *Aplysia californica*, and *Ciona intestinalis*. Tree was rooted with the NR 2 family. Values above branches indicate percent of 1000 bootstraps. Click here for file

Additional file 6 **Intron-exon structure for seventeen *N. vectensis *NRs**. White boxes indicate exons with coding sequence, gray boxes are exons or portions of exons containing the untranslated regions, and lines between boxes indicate introns. Click here for file

Additional file 7 **Exon boundaries (vertical bars) for two sets of inferred cnidarian-specific NR radiations**. (A) NvNR8 and 9, TLL-like genes, share 2 of the 3 exon positions. We could not compare the first boundary in NvNR9 with NvNR8 because we do not have corresponding sequence data for this portion of the protein. (B) Four NvNRs inferred to be duplications related to COUP-TF. NvNR12, 13 and 14 each has 3 exons with two conserved splice sites in all three genes. These two positions are also conserved in NvNR11, but this gene has two additional exon boundaries without conservation in the other three NRs from this subfamily. Click here for file

Additional file 8 **Primer sequences for amplifying pieces of NvNRs for cloning and for qPCR**. Table of primer sequences. Click here for file
