# Supplementary material for: Nuclear receptor complement of the cnidarian Nematostella vectensis: phylogenetic relationships and developmental expression patterns
Source: BMC Evol Biol. 2009 Sep 10;9:230. doi: 10.1186/1471-2148-9-230 (PMC2749838; doi:10.1186/1471-2148-9-230)
Supplement: Additional file 1 — N. vectensis deduced amino acid sequences used for phylogenetic analyses. List of amino acid sequences. [file 1471-2148-9-230-S1.doc]

Additional file 1. *N. vectensis* NR deduced amino acid sequences used for phylogenetic analysis.

>NVNR1

MDKTFLVTSSQRVCAICGDRSSGFHYGVQSCEGCKSFFKRTVQKQLQYACVESKSCQIDKNNRIRCQYCRFQKCLALGMLKEAVREDRAPGGRPRIKSLIGLKENAETFVSSELIIQLIQARPDAIPKRRPDYLELGLNDMCPLNPVEAIMELTLQEVEHVINWAKRVPGFCDVDKEDQISLLSEGLLEVLILRICQRSTPHQGAVMVAKDTLLSPDNVFNIVLEQWAGQLSCFAYKLQSLQLDMAEFACVNAIMLFEQETSGGLRNRDLVDFVLNRTLDALRDYIKSSYPEKPSRFAHILLKLPTLRDMATRMAEECLFAQSLLHLAIPQLTSRMIELNHGRP

>NVNR2

MASEEKCAVCGDESSGYHYGAYTCEGCKSFFKRTVQRGLVKKYSCVRSNQNQCPMDKNSRAKCQACRLQRCFDAGMVLNGVRKEKKRGGRSFYTLKSDPNETPTTEKLPSPTPSSPDTAHIDSTSISPLLRDLLASNPCV VPKGESLKDIEESFKEGKKFLLIRLSNAITKELHIIVDWAKLVPGFKDLCQEDQITLLTAAGMELVVFRVIFRSIPLPNYIYMTTTTCLQREGCYEILNKEIVDMLLDVVDRLRSVGLDTVEFACLMAILLADPENPGLLEKPKVEELRTSFLSGLKLHVETVYPSQPERLAKILLRLPALRDVCTK

>NVNR3

MLLCVVCNDKATGYHYGVFTCEGCKGFFKRTVQKQLEYTCRGNQDCDINQHTRNRCQYCRFEKCVKSGMLKQAVRDDRTPGGRHRHASLQDHRQKKQKLQGKAPKELPQNGLSSPEGPDTPEGVVSKEDENEFLIYIQQLRHDVDVIPDSPVKPPEGSPAAGFSVDKLMQYGYQELYQVIQWGKNVPGFRELKLEDQITLLKTSFMDLNVFRLAYRSICCDPDSLMFAKGIILNKPQCLEMGWSMDLTETTLEFCAKLRNLNMDVNEFSCLSGLVLLSPDAPGVVDKDKVTELQTNVTSCLRDYEMYRYPSKSNRLGKLLLCLPSLRAYSEKALENYVTLEFFGKLDMPPLVAELLE

>NVNR4

MTANLVNGVKQESGGGDDGDGNGACLICADRATGKHYGASSCDGCKGFFRRSVRKNHVYSCRFQRNCVINKDKRNQCRFCRLRKCFRAGMKKEGTSAVQNERDSISKKPKEEKKYGLTTQSLLSAEILSRPASSPPESICLEKEANYSDICESMRQQLLILVEWAKHLPCFCELPLDDQVALLRAHASEHLVLGVSRRSLNLKDILLLGNDLVIPRQAGDTEVRKIATRILDEIVLPMRQLNIDDVEYSCLKAIVFFNPDAKGLGDSQKIKALRFEIQTTLEDYISDHQYECRGKFGEILLLLPTLQAIALQMVEQIQFARLFGVAKVDSLLQEMLLGGTNITEQLDAASLSLSQPPPDGAVSPTEQVLSSPPPEQLFAPSLTPVISGTPNLGMTTPVSQMSDKISASQPEQQFTSQMALMNTAPAPSLPTLSSEDISRETSVTRSQPKRTATRQQYFRQEQCSSVAKRERFDT

>NVNR5

GRLLDIPCKVCGDRSSGKHYGIYACDGCSGFFKRSIRRNRSYTCRATNGKGNCPVDKIHRNQCRSCRLKKCFDVSMNKDAVQHERGPRSSTLRKQKMLKEAQERLELVSVAHAPQGPSGFINTLLAAEPRMDGCGMAVNDFDLEYKPIRLQSDMSVPMYYSSPESVCEAAAKLLFMSVKWARNIPSFMSLPFRDQVILLEEGWRELFLLGASQWSMPLEIAPILAASGMHVDNTPPEKIVDVMATVRTLQEAVNKFKAFGVDSTEFACLKAIVLFKPAACGLKDPEQIETTQDQAQLMLGEYIRSTYPTQVARFGRLLLLLPALRRVSALAIEELFFKKTIGTVPIERLLSDMFKNEQ

>NVNR6

MNDSHDPRTDSKPCRKRIQDDQVCVVCGDQSTGKHYGVFACDGCSGFFKRTSRRLEPWVCKAQNACSVKTTSRNECKACRMKKCVEVGMKFDGIKHSDFHVSTQTPLHQQATEDSNYPSGTDFDTINPSNFSSTISESSHSASPYEELSTPNRPTISYVQQVSSKNIQDAATRLLSASIRFARNVPCFTRLPFRDQIILLEEGWKELFLLDAAYWALPLEIASLLAVTGGCHGDSYRHKASEIKLLQELLARLRSFQMDLNELACLKAIVLFRPETKGLKDSDQVDKIQDHIQLLLAHHTMTKHPTHPSRFGKLLLSISPLHSLAEKPIEDVLFRKTDDKDIFESVLSQLMDNSC

>NVNR7

MDKKQEILCKVCGDISSGRHYGVYTCDGCSGFFMRSVRRDMVYTCKGNGGCTVDKKRRNQCQACRFKKCLEVKMNRFAVQQERQPNSTRVMKPHLDEYGLKGLNNEFLASLIVAEPCRDAVYRTSQGLNLPYLQQDVASPLFCSSPDALFESAARLLFMSVKWARNIPSFVNLPFRDQVVLLEEGWRELFIMGAIQWNLPLEVAPLLAAAGMHVDNTPAEKIVATMADIRKLQEIGSRFRALQVCEAEFACLKAIVLFKPDLRDLRDPQQVECYQDQAQIMLGDYIKRQFAGQQVRFGKLLLMLPSLRLVRNKTIEELFFRQTIGSVAIESLLCDMFKSS

>NVNR8

TVQCRVCGDRASGKHYGVMTCDGCRGFFKRSVRRNLAYQCKEKNDCPIDVARRNQCQACRLRKCFEMNMNRDAVQHERAPRTSQIRQNSQSKPLKRKHISIENMSADQIKTKSDKRAYQQVDSRSSGFEKPIDDMENVKCLPPVSIASNLTISPTPHSVGIPVVYMDMMYESAIRVLYMTVKWVRNIPTFLDLPFRDQAILIEESWSELFILSLSQWDMAVDLSSLVSAAYPQQMGLSPGHKSDRTPGSMSDIRNLQTVVSRLRTASVDQTEYACLKAIVLFKPDIRGLRLWSHVEQLQDQAQCMLGEYELQHYPDQVSRFGKLLLSLPSLKSIPPKAIERLFFSGTLDNIPMERLLADMFKSS

>NVNR9

MMDMGVVGNSDEGLTVDRKPLVLCRVCGDRSSGKHYGVFTCDGCRGFFKRSIRRNLTYHCKELGKCVVDVARRNQCQACRLKKCFEVQMNKDAYPQSDSGSTPPSSPPYHGQNNNRSPDSSKKHSFMSIASLIDTKENQDGKKDLNKEDRPDGVPTQRSTDMCPGWTFPTGYQTAPQDSIYESAVQLLYMSVTWARNIPTFLDLPFRDQAILLEEGWSELFVLSSAQFSLPLDMGPLLSAAGLQVDKAPTDRIVAGMADIRLLQNIVTRFKRLQIDSTEYACLKAIVLFKPDLRGLRAPQLVERLQDQAQSMLGEYCRSQYPDQQVRFGKLLLMLPSLKTVSPKMIEDLFFRGTLDNVPIERMLCDMFKSS

>NVNR10

MEVAPVTTWTRDSTETPEDSEKNVQVECAVCGDKSSGKHYGVFTCEGCKSFFKRSVRRNLTYTCRASRDCPIDQHHRNQCQYCRLKKCLKVGMRREAVQRGRIPAAQTPTQNAALPGINGDGSTNGHSYLSGFIALLLRAEPYPTTRFQQGLNMPCGIMGIENICELAARLLFSAVEWARNIPFFPDLAVTDQVALLRLVWSELFVLNAAQCPMPLQVAPLLATAGIHSNHMSPDRMVSFMDNIRIFQEQVEKLRNLHVDAAEFACLKAIVLFTSDASGLTDPQYIESLQEKTQCALEEYTRNQYPNQPTRFGKLLLRLPSLRSISSSVVEQLFFVRLVGKTPIETLLRDMLLSGTPTTWPYLPCS

>NvNR11

MNGSVFWSPDGGVDNDPDSPASSSSKPLYIDCAVCGDKSSGKHYGVYTCEGCKSFFKRSIRRSLSYSCRGVRNCPVDIQNRNQCQYCRLKKCLKVGMRKEAVQKGRIPSTHPDVGPLSVSMVEMNGHQSFYSSYITLLLRADTIARYQQSLTLPCNINGLENTPELAARLLVSAVEWAKNIPFYSDLPLPDQAVLLRSCWSELFTLNAAQHCSPFHISPTLTSNSSGFAGNGGGYLNTRVMSAFDCQNNNMKLFEEQVEKLKNMHIDSAEFACLKAIVLFNPDSQGLSEPAQVENLQDRTQSALEDYIRTQYPNQTTRFGKLLLRLPALRLLRPVSVENLFFSRLSMGNTVDSLLNDMLLSGLGGGVVPWLPGPSPPLNCTTSNMNVITQM

>NvNR12

MPKHKTDERVACAVCGDKSTGKHYGVSTCEGCKSFFKRTVRNNTNYTCRGQNTCAIDRNSRSRCPSCRFQKCLSTGMKKEAVQTTKLPPFPALQFPFYGDVNTMYAQTMFPLTLFQSPFNAPLTFPMGMVPLNQNRTDVAYELAANVLFAVVDWARKLTTFNNLMDSDQITLLKMAWTDLFLLEASRSPLQLYVQQMYATINAQTKQLSMEVIVKRMEYARLFQEQAERIRNLGMDMTEHFHLKCIVLFRADGSLINQPRQVEVLQDTSQSSLEQYIRSQYPSQPTRFGKLLLMLSSLRKVESTVIEQLFFADVLRGASMGEVLKKMLTTGNQSPTTLAAALANGKGSPMS

>NVNR13

MSSWSQMHEVYWYDNPDKTIECAVCSAPSSGRHYGVFTCEGCKCFFNRTVRYKLTYICEGSGSCRVDKQNRTQCQACRFKKCATVGMRREAIRRGRPTKYSYISRSSKSFTPQYDLISVLTQLERSIRPPVPYPSSGLQSSPVSMYHRVCSILVSTLDWSRRVPMFANLDVCEQYSVLRSRWCEMLIVSAAQYEVHIDGIPLAYEVEMNPGFCNEKQIQLKRSLRNFQESVWRLRGLEEAEYACLKTIILFSPDASEGPFVQEFESLQELVLSALDRFCRARFPEEPSRYGKVLLKLMSLKSVIAEDIETLVFSKLFPHSSVSGIIRNHLVSDVTSAPESMSPAKTSPVNQ

>NVNR14

MEICSVCGDHSTGKHYGANTCEGCKLFFKRSIKKRLYYTCRVAGCCPVNKRYRNSCQFCRMRKCLAVGMKREAVQQARAQDFEKARRRQKRQQHKGEIRKEADFATLSDFVFHLQAVEPYCRRQESESKTPESTDCGSEEISREKSPAEKTSEMAARLLFMTAHWAKKVKHFSELSHFDQVTLLRENWSKVFIINLVQWAMPFEIAPIVSDIVEKTPGQHLDKVLHTMGKLNEVVFKLVQLQLSRAEFSLLKALALFNPDTEQLADAVQIQAVQNKTQNALEEYIRVHYPQTPNRFGQVLLRLTALGAVECKIIEHVFFNKLLGSTSIYTLVDDILLSKELNSE

>NVNR15

MTTQSIPICLVCNDRASGRHYGVMTCEGCKGFFKRSVRRNMVYHCMFRETCIVDKVLRNRCQKCRMDKCLAVGMQKSGKIFYAKINVEARNPEQYASAMAEFYPKLFLFFSAVQYERRPLSCSTRTSTLSSSSSHSSTLNSPVMDKSIIGPEQVPLSLSLLNIECPKDSIEYLYEVSTRLVLRSIDWARGIPAFLSLPSLDQITLLEASWSGIFCLGVVQCTEIFPLDVLSRLVKKKFACADDDDAKHDGRGVAFVSKLSLGQRLTAMRNLISSMRRMSVDATEFAYLKGLLLFDPDKATVNIKQITDLQETISESLQVYLSKRQPPETTRFASLLLRLPAARMLTHEVLEEVFFPPVFNNSKILLLIAGAVGDAGST

>NVNR16

MVAIPKPIIWGNCKVCGDNGTGKHYGVVACEGCKGFFKRTVRKNLIYTCRGSNDCFIDKVHRNRCQKCRFVKCLTAGMKKEAVQCERKPLAASQLVRQQETPVITSTEEREDRGYASESNSEQSLSPPAVRNNPRQEHREHVAEILVLQTEFKVNVPSLAGVQVFSMDYVYEFATRLLFVSIDWTQSISAFRALHKCDQIALLCKTWADIFLLGVAQSISNFPLSPLLSLAAKDIQQSEAQDPKPPKNIPGQKNTFEKIIAIKDVMFSFEKLNLDATEFAYLKAVVLFNSSDPYTCVQDQKQVDKLQEQSHCGLKSYIDSKYPIGNSGRFAKILLRLPSLHLIDRFDVEELFFAPLLGGVKIESIMEKIILRSSTSSF

>NVNR17

MDGNKPRKPSELEGREFQDKRRANGMVSTEDKIFPRPLAVTQGLSPLLFHQPMLPLHRQQPGMQHFHAPYLTQASLFFSPQLINSHQLPYSQQQIISQRGGVIHQVAPLVRLTTEERLQQELNGSSREQYSKDRPISSTSDRSQGSPEAPKSQPMKVIHQVSRDEKDYEQDDSPLSQPMSCDSMPSTPKSEASPQAPIKSPETPPSFSAASPRPVVNEICAICLDKATGQHYGVTSCEGCKGFFKRSVQNKKDYTCRNLTKDCPIDKRHRNRCQYCRFQKCIQAGMIKEAVREDRTPGGKHKSSLLRTKSVQDAIPCKMRRTSSTSSCSSVLSSATTAAQVKPTKEYIEVIDELIGIYKLDNQSCDDCPGCKETGMKEKTLAHLTQLAEQQLVRCASWFKHLKLLKGICELDQQTLVTNVWVELMLANLIKESENLENKAKLCDGQILDFETAEITGIGDILQRVVQMAAKFREFQLEKVEIVCMKMIILLNPDLPGLQNQQLIEQLQDKVHSALQEHINLAFPREPNRFGNILLRLPELRSIGTKSLERLFMLNLTGQIHPSTSLSDLLHTGKR
