# Supplementary material for: Nuclear receptor complement of the cnidarian Nematostella vectensis: phylogenetic relationships and developmental expression patterns
Source: BMC Evol Biol. 2009 Sep 10;9:230. doi: 10.1186/1471-2148-9-230 (PMC2749838; doi:10.1186/1471-2148-9-230)
Supplement: Additional file 8 — Primer sequences for amplifying pieces of NvNRs for cloning and for qPCR. Table of primer sequences. [file 1471-2148-9-230-S8.doc]

Additional file 8. Primer sequences for amplifying pieces of NvNRs for cloning and for qPCR.

| Nuclear Receptor | Cloned Fragment Primers (5’ – 3’) | qPCR Primers (5’ – 3’) |
| --- | --- | --- |
| NvNR1 | GAACGCTGAAACATTCGTCTCG  AAGCATTCCTCTGCCATCCTGGTC | ATAGGGTGCCGGGATTTTG  AGGACCTCAAGCAGACCTTC |
| NvNR2 | ACTGCAGCGTTGTTTTGATG  CCGCAATCTGTCAACAACAT | GGGTCAGGTTTTAGGGTGTAG  ACTGCAGCGTTGTTTTGATG |
| NvNR3 | CGTGGAAATCAAGACTGTGACATC  GCATCAGGACTTAGTAGCACCAAAC | GCAAGCTGTTCGTGATGATAG  GACAGGCCATTTTGTGGAAG |
| NvNR4 | CTTCTTATTTTGGTGGAGTGGGC  GCAGTTCTTTTTGGTTGTGAGCG | CACTGCAGATGGTGGAACAG  CTCGGTTATGTTGGTGCCTC |
| NvNR5 | CGACAAACGGAAAGGGGAACTG  AATCGGGCTACCTGTGTGGGATAG | AAATTCATCGCAACCAGTGC  TTCGTAGGGTGGAGCTTCTG |
| NvNR6 | TTCATCAGCAGGCAACCGAG  TGGGTGGGGTGTTTTGTCATCG | AGGGACCAAAGGCCTAAAAG  GCAAGAAGAAGCTGGATGTG |
| NvNR7 | GACGGTTGTAGCGGTTTCTTTATG  CCCAAATCGGACTTGTTGACC | CTTTTCAAGCCAGACCTTCG  GCCTGGTCTTGGTAGCATTC |
| NvNR8 | TGTCAGCCGATCAAATCAAA  ACGAAGACCCCGTATGTCAG | GGGTTCGTAACATTCCAACG  CTCCGGCTCTCCTCTATCAG |
| NvNR9 | GCAAGTGTGTTGTGGATGTAGCC  CAGTCGTTTGAACCTCGTGACG | ACTGGGCAAGTGTGTTGTG  GTTGGACCGCGTCTTTATTC |
| NvNR10 | CACAAACGATGGCTCAAGGAAC  GGACGATTGCTTTCAGACACGC | AACAAGCGGACTCAAACCAC  ACCGCACACTCAACTTGGAC |
| NvNR11 | GCGACAAATCTTCGGGGAAAC  AAAACAGGTTCTCAACTGACACCG | AAGGAAGCGGTACAGAAAGG  CTCGCAAGAGGAGGGTAATG |
| NvNR12 | AGAAATAGCAGAAGTCGTTGCCC  GGAGAAGTTTGCCGAATCGC | AGAAGCGGTCCAAACCACC  GAACAGGGTCAGCGGAAAC |
| NvNR13 | CCCAGACAAGACCATCGAGT  ACCAACGAGAACGCAGTACC | GTTGCCGTGTTGATAAGCAG  ATGGCTTCTCTCCGCATTC |
| NvNR14 | AAGTGCTTAGCCGTCGGAATG  CTTGTCCAAATGTTGACCTGGTG | CGTCGGAATGAAGAGGGAAG  GCAAAGTCAGCCTCTTTTCG |
| NvNR15 | AATCCATCCCCATTTGCCTG  CCTTCCCAATCTTATGTCACGC | AAAATCCGCTGTCCAGTACG  AAGCTCAAAGGCACTTGCTC |
| NvNR16 | CAGGAAAACACTACGGAGTCGTTG  GGGGAGTCTGAGGAGAATCTTAGC | TACATGCCGTGGAAGCAATG  CTTCATCCCCGCAGTCAAG |
| NvNR17 | GCTGTGAAGGCTGTAAAGGGTTC  GGGTGTATCTGTCCTGTGAGGTTC | GTGCCAGTACTGCCGTTTTC  CGCTTCCTTTATCATTCCAG |
